# Evaluating the metapopulation consequences of ecological traps

**DOI:** 10.1098/rspb.2014.2930

**Published:** 2015-04-07

**Authors:** Robin Hale, Eric A. Treml, Stephen E. Swearer

**Affiliations:** School of BioSciences, University of Melbourne, Parkville, Victoria 3010 Australia

**Keywords:** dispersal, habitat selection, human-induced rapid environmental change, natal habitat preference induction, source–sink dynamics, topology

## Abstract

Ecological traps occur when environmental changes cause maladaptive habitat selection. Despite their relevance to metapopulations, ecological traps have been studied predominantly at local scales. How these local impacts scale up to affect the dynamics of spatially structured metapopulations in heterogeneous landscapes remains unexplored. We propose that assessing the metapopulation consequences of traps depends on a variety of factors that can be grouped into four categories: the probability of encounter, the likelihood of selection, the fitness costs of selection and species-specific vulnerability to these costs. We evaluate six hypotheses using a network-based metapopulation model to explore the relative importance of factors across these categories within a spatial context. Our model suggests (i) traps are most severe when they represent a large proportion of habitats, severely reduce fitness and are highly attractive, and (ii) species with high intrinsic fitness will be most susceptible. We provide the first evidence that (iii) traps may be beneficial for metapopulations in rare instances, and (iv) preferences for natal-like habitats can magnify the effects of traps. Our study provides important insight into the effects of traps at landscape scales, and highlights the need to explicitly consider spatial context to better understand and manage traps within metapopulations.

## 1. Introduction

Humans are altering ecosystems at significantly faster rates than natural forms of environmental change. This is referred to as human-induced rapid environmental change (HIREC) [1], and it leads to profound changes to habitat quality and quantity. Whether dispersing animals can continue to adaptively select suitable habitats in the face of HIREC is an important question, particularly if the cues used by animals during habitat selection become uninformative of habitat quality. This breakdown between habitat preferences and quality can cause ecological traps, when animals select habitats that provide fitness outcomes inferior to other nearby habitats [2–4].

Ecological traps were originally described following studies with birds [5], but occur across a wide range of taxa (for examples, see [3]). Perhaps the most compelling case is the attraction of insects to artificial sources of polarized light (e.g. roads) and their subsequent death upon landing [6]. Traps can also have sub-lethal effects; for example, red-backed shrikes in northwest Europe prefer open areas created by farming, where their reproductive performance is lower than in nearby forests [7]. Given the mounting evidence across taxa and ecosystems, ecological traps are likely to increase the risk of extinction and loss of biodiversity, with important implications for conservation and management [8]. This has motivated efforts to better understand how traps form and to describe their potential effects (e.g. [2–4,9–12]). Much of this effort, however, has been at the scale of local populations, such as testing which of a few habitat patches may be traps (e.g. studies within [4]), or modelling the effects of traps in landscapes with only two habitat types (i.e. ‘low’ and ‘high’ quality). Furthermore, while the impacts of traps will depend on their severity (i.e. attractiveness, fitness costs) [11,13], the local dynamics of traps play out within the context of landscapes that may have a mosaic of different-quality habitats, especially as they are modified under HIREC. There are also reasons why inferring the wider-scale consequences of traps from local studies may be problematic, such as if habitat preferences are evaluated at a small number of locations that are poorly representative of those available [14]. This suggests that further insight could be gained by evaluating ecological traps within a broader, regional context.

The metapopulation concept is a standard ecological paradigm for exploring the dynamics and evolution of organisms in spatially structured landscapes [15]. In most ecosystems, local patches vary in habitat quality, which often influences vital demographic rates and thus the persistence of local populations [16,17]. This can lead to source–sink dynamics, where poor-quality habitats cannot support local populations without replenishment from other patches [18,19]. The concepts of ecological traps and source–sink dynamics are related; a sink is analogous to an ecological trap if it is preferred over other patches, but source–sink theory does not incorporate the potential for maladaptive habitat selection [13], a key characteristic of a trap. Whether ecological traps occur, and whether their presence reduces the growth rate and/or persistence of metapopulations, will ultimately be determined by how dispersal and habitat selection, and their associated fitness consequences, are altered across landscapes. At present, our ability to assess how traps may compromise metapopulations and how their effects can be best managed is hindered by a limited understanding of how traps impact spatially structured populations across a landscape.

### (a) A framework for assessing the landscape scale consequences of ecological traps

We develop a conceptual framework integrating metapopulation theory, movement ecology and sensory biology to assess the consequences of ecological traps for metapopulations within spatially explicit landscapes. We propose that the factors likely to determine the consequences of traps for metapopulations can be grouped into four main categories as follows.

#### (i) The probability of encounter

This will depend on both the physical environment and the life-history traits of organisms. Animals will encounter traps more frequently when they represent a larger proportion of available habitat. The underlying topology of the landscape is likely to affect metapopulation persistence when traps are present, as has been demonstrated in studies without traps [20,21]. The characteristics of trap patches relative to other habitat patches will also probably be important. For example, large traps may represent larger ‘targets’ for dispersers, or traps that are isolated from other patches in the network may be encountered less frequently [22–24].

Their dispersal and perceptual ability will affect the probability that animals encounter traps (electronic supplementary material, appendix S1). Range-restricted species may be highly susceptible [8], whereas vagile species may encounter traps more frequently but have greater capability of ‘escaping’ by moving to more favourable habitats. Migratory species with obligate habitat transitions may be particularly susceptible. Finally, animals with a larger perceptual range [25] may be attracted to habitats from greater distances, potentially increasing the frequency with which they encounter traps.

#### (ii) Likelihood of selection

Animals may exhibit a preference for traps (a ‘severe’ trap), or equally prefer traps and non-traps (an ‘equal-preference’ trap) [3]. In both cases, animals will select traps more frequently if their dispersal is uninformed (i.e. an imperfect knowledge of the environment) and they rely on indirect habitat selection cues acting as proxies for habitat quality [8] (electronic supplementary material, appendix S1). Animals that use multiple cues (either simultaneously or sequentially) to locate and assess habitats (e.g. [26]) may be less susceptible, as they will require multiple stimuli to incorrectly indicate high habitat quality. Physiological changes to dispersers may also increase the probability that animals will select traps; animals in poor physiological condition or under tight time constraints will generally become less choosy in terms of selecting mates or habitats (see [27] and references within), or more likely to choose poor-quality habitat [28].

Natal experience influences habitat preferences for a wide range of taxa including insects, fish, mammals and birds [27,29,30], and almost always leads to a preference for natal-like habitats, known as natal habitat preference induction (NHPI) [30]. Although NHPI could weaken the effects of traps [11], we suggest that it could equally cause them, if animals raised in poor-quality natal habitats select similar ones later in life. For example, common loons in Wisconsin select lakes similar to their natal site in terms of pH and size, not necessarily the large, high-pH lakes that produce more and fitter offspring [29].

#### (iii) Fitness costs of selection

The consequences of traps will depend on which components of fitness are reduced and by how much. In the most extreme case, traps will result in mortality (e.g. [31]), but other non-lethal endpoints such as reduced reproductive success are also possible (e.g. [32]). These various effects may have different consequences for metapopulation growth and persistence (e.g. [33]).

#### (iv) Species-specific vulnerability to fitness costs

Life-history traits will be an important determinant of an animal's vulnerability to traps [8,11]. In particular, traits from the ‘slow’ end of the ‘fast–slow continuum’ [34] are likely to increase long-term vulnerability (e.g. delayed sexual maturity, low fecundity and long generation time; see electronic supplementary material, appendix S1). Animals with rapid adaptive potential may be able to respond quickly and escape the effects of traps, while those with low capacity for learning, slow rates of evolution or a lack of behavioural adaptations to change will be most susceptible [11].

We illustrate this framework using a metapopulation modelling approach, and compare how different characteristics of ecological traps can affect metapopulation growth and persistence. As identified above, the impacts of traps will be dependent on a large number of factors. We selected a subset of these, which we predicted *a priori* to be important, and examined how they affected the consequences of traps for metapopulations to evaluate evidence for the following six hypotheses: (i) the effects of traps will be more pronounced when they represent a larger proportion of available habitat; (ii) the chances of an animal encountering a trap will depend on their dispersal ability or perceptual range; (iii) severe (i.e. preferred) traps will reduce metapopulation growth rate and persistence more than equally preferred traps; (iv) animals that exhibit NHPI will be more likely to select traps; (v) reductions in breeding fitness and mortality will have differential effects; and (vi) animals with life-history traits from the ‘slow’ end of the fast–slow continuum will be more susceptible to traps.

## 2. Material and methods

### (a) A modelling framework to examine the influence of traps on metapopulations

To accommodate the full range of landscape-, trap- and species-level variation, we used a network-based landscape representation [35] with species-specific attributes (dispersal, fecundity, survival) coupled with a gravity model [36,37] to parametrize habitat selection. Patch attractiveness is a key element determining how a habitat patch will be perceived by an individual, and we implemented this process with a production constraint gravity model [36] of dispersal. With this approach, the functional connectivity between any two habitats is a function of (i) geographical distance, (ii) dispersal capacity, (iii) reproductive output of the source patch, and (iv) size and attractiveness of the destination patch. All metapopulations varied in the number, placement, size and quality of habitat patches containing subpopulations. Species-specific attributes, unique to each metapopulation simulation, included dispersal ability (negative-exponential decay function), perceptual range, habitat preference, survival and fecundity. From this initial landscape, a proportion of habitat patches was selected and converted to traps by decreasing fitness (i.e. survival and/or fecundity) within these patches and increasing their attractiveness (‘realized traps’; electronic supplementary material, appendix S2). A natal preference penalty was used to modify the dispersal probability, redistributing individuals to patches with similar qualities. Landscapes often comprised a mosaic of different habitats that vary in location, size and quality [38,39]. When animals have imperfect knowledge of the environment, they can make suboptimal habitat selection decisions [25], resulting in naturally occurring patches with trap-like consequences (e.g. low-quality yet large, and therefore ‘attractive’, patches). Ecological traps are defined as arising from changes to the attractiveness of and/or preference for a particular habitat [3], so we did not code these trap-like patches (whose attractiveness and/or quality had not been altered) as ecological traps but treated them as natural phenomena of heterogeneous landscapes (electronic supplementary material, appendix S2). However, because the majority of simulations contained trap-like patches, we also evaluated their overall impact on metapopulations (electronic supplementary material, appendix S2). All model parameters are outlined in table 1, and further details of these and the modelling approach more generally are provided in electronic supplementary material, appendices S2–S6.

**Table 1.** Description of variables included in the model and descriptors of their characteristics. See electronic supplementary material, appendices S2–S6 for further details.

	parameter	description	range
landscape configuration	number habitat patches in landscape (N)	limited to 50 for computational efficiency	[3,50]
	minimum quality of habitat patches (MinQ)	patches were randomly assigned a quality < MinQ	[0,1]
probability of encounter	trap proportion (T.pro)	proportion of patches in the landscape that are traps	[0.1,1]
	dispersal capacity (Disp)	the relative distance at which the probability of dispersal is 0.05; using a negative-exponential function, *p_ij_* = exp(*θ* × *d_ij_*), where *θ* is the decay coefficient and *d_ij_* is the distance between patches	[0,1]
	perceptual range (Pr)	the perceptual range of a patch is a multiplicative function with patch size, quality and Pr	[0,5]
the likelihood of selection	attractiveness of traps (T.att)	attractiveness of traps is increased by T.att	[1,10]
	preference for natal-like habitats (Np)	dispersal between patches decreases proportional to the difference in quality times Np	[0,1]
the fitness costs of selection	trap survival penalty (T.surv)	survival in traps decreased as Surv × T.surv	[0,1]
	trap fecundity penalty (T.fec)	fecundity in traps decreased as Fec × T.fec	[0,1]
species-specific vulnerability to these costs	fecundity (Fec)	the number of offspring per unit area as a function of quality: Fec × *q*_i_	[2,100]
	survival (Surv)	the survival of adults per unit area, as a function of quality: Surv × *q*_i_	[0,1]

The consequences of patch-level demographics and animal movement within each metapopulation were quantified by calculating the metapopulation mean lifetime (MMLT) and metapopulation growth rate (*λ*_M_). The network-based MMLT calculation [20,40] accommodates habitat networks consisting of patches of variable size, quality, spacing and a stochastic extinction likelihood, in a computationally efficient approach. Simply, the MMLT is a function of three network characteristics: the dispersal network structure, extinction rates of local populations and the size of habitat patches. We used the Kininmonth *et al.* ([20], eqn 10) approach for calculating MMLT for all metapopulations, using constants for the species-specific minimum patch size coefficient (*ɛ* = 1.0), extinction area exponent (*η* = 0.5) and the minimum number of immigrants for successful colonization (*μ* = 2.0). As a result, the patch-level extinction risk in MMLT is a function of its area and quality. Similarly, we used a metapopulation growth rate calculation sensitive to the spatial structure of the dispersal network, as well as patch-level demographic potential and its contribution to other patches [41]. This network-based *λ*_M_ ([41], eqn 13) is dependent on the dispersal network and the individual patch attributes of area, fecundity and survival. The MMLT and *λ*_M_ quantify slightly different (extinction risk and growth rate, respectively), yet complementary characteristics of the metapopulation.

To make meaningful comparisons across all models, we quantified the relative impact of traps on *λ*_M_ and MMLT by evaluating each metapopulation both with and without ecological traps. These paired models were used to calculate the relative impact of traps on the metapopulation: *λ*_MImpact_ = (*λ*_MTrap_ − *λ*_Mnon-Trap_)/*λ*_Mnon-Trap_) and MMLT_Impact_ = (log_10_(MMLT_Trap_ + 1) − log_10_(MMLT_non-Trap_ + 1))/log_10_(MMLT_non-Trap_ + 1). Larger negative values in *λ*_MImpact_ and MMLT_Impact_ indicate stronger detrimental consequences of traps on the metapopulation. Owing to the unknown prevalence and strength of NHPI, we analysed each metapopulation pair with and without the natal preference penalty. There was high concordance in metapopulation impact between analyses with and without NHPI, so only results with NHPI are presented.

### (b) Model sensitivity analysis

We used a variance-based global sensitivity analysis (SA) framework [42,43] to evaluate the consequences of traps. For computational feasibility, we implemented a non-parametric SA based on a series of meta-models [44–46] using all input parameters (R package CompModSA with ‘sensitivity’ function). A suite of 3000 parameter combinations generated with a Latin hypercube sample (LHS) scheme [45,47] was used to build each meta-model. Each parameter combination resulted in a unique metapopulation model realization.

Owing to the complexity of the model, expected high-level interactions among parameters and nonlinear responses, we evaluated several meta-models to examine consistency in emergent patterns [45,48,49]: generalized linear model (GLM), quadratic response surface regression (QRS), recursive partitioning regression (TREE) and multi-variate adaptive regression splines (MARS), all implemented in R (GLM with the MASS package, all others with the CompModSA package). These meta-models were chosen as each is expected to perform differently depending on the unknown structure of the response surface (see electronic supplementary material, appendix S2 for meta-model comparisons). For the GLM SA, we calculated the main effects and two-way interactions on the standardized data and visualized the sensitivity of response variables by plotting the effect of one standard deviation change in each parameter on the response [49]. For the non-GLM meta-models, the total sensitivity index, 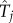 [48,50], was used to quantify the relative importance of all input parameters to the relative changes in MMLT and *λ*_M_ owing to traps (MMLT_IMPACT_ and *λ*_MIMPACT_, respectively). This index provides a single number summary of the overall importance of each parameter and should be interpreted as the total proportion of the variability in the response surface that is due to each parameter, including all interactions with other parameters [48]. Standard bootstrapping (10 000 samples) was used to create confidence intervals around the mean sensitivity index value.

## 3. Results

**Figure 1.**
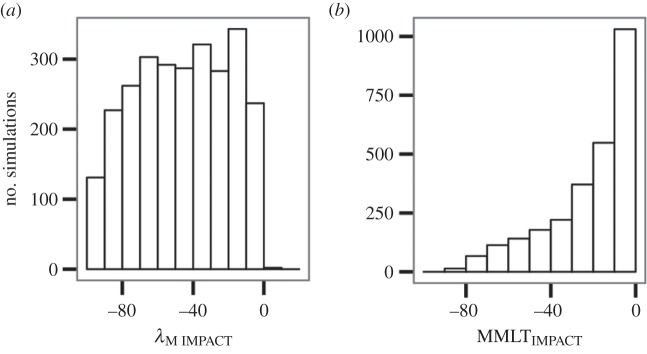
The effects of ecological traps on differences in (*a*) metapopulation growth rate (*λ*_M IMPACT_) and (*b*) mean metapopulation lifetime (MMLT_IMPACT_) between trap and non-trap metapopulations (*n* = 2688 simulations).

After screening the 3000 unique parameter combinations to remove scenarios where the non-trap metapopulation had a decreasing growth rate (*λ*_M_ < 1) and those lacking realized traps, 2688 (90%) remained. In almost all cases, traps had negative effects on metapopulations in these models (figure 1; median *λ*_M IMPACT_ = −45.10, median MMLT_IMPACT_ = −14.80). However, in some rare instances, they were beneficial, resulting in positive *λ*_M IMPACT_ values (figure 1).

**Figure 2.**
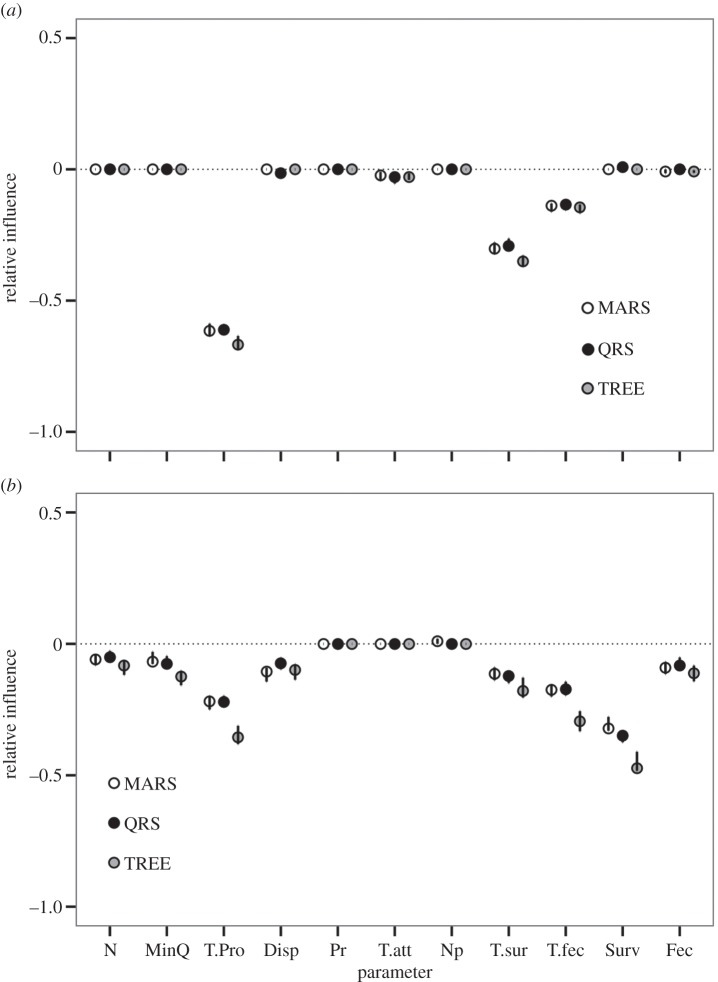
Results of global sensitivity analysis describing the relative influence of variables (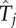) on differences in metapopulation (*a*) growth rate (*λ*_M IMPACT_) and (*b*) mean lifetime (MMLT_IMPACT_) between metapopulations with and without ecological traps. Overall fits (*R*^2^) ranged from 0.83 to 0.94 across all models. Negative 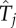 values indicate that as the parameter value increases, the severity of the impact of traps increases (becomes more negative). Model parameters are defined in table 1.

**Figure 3.**
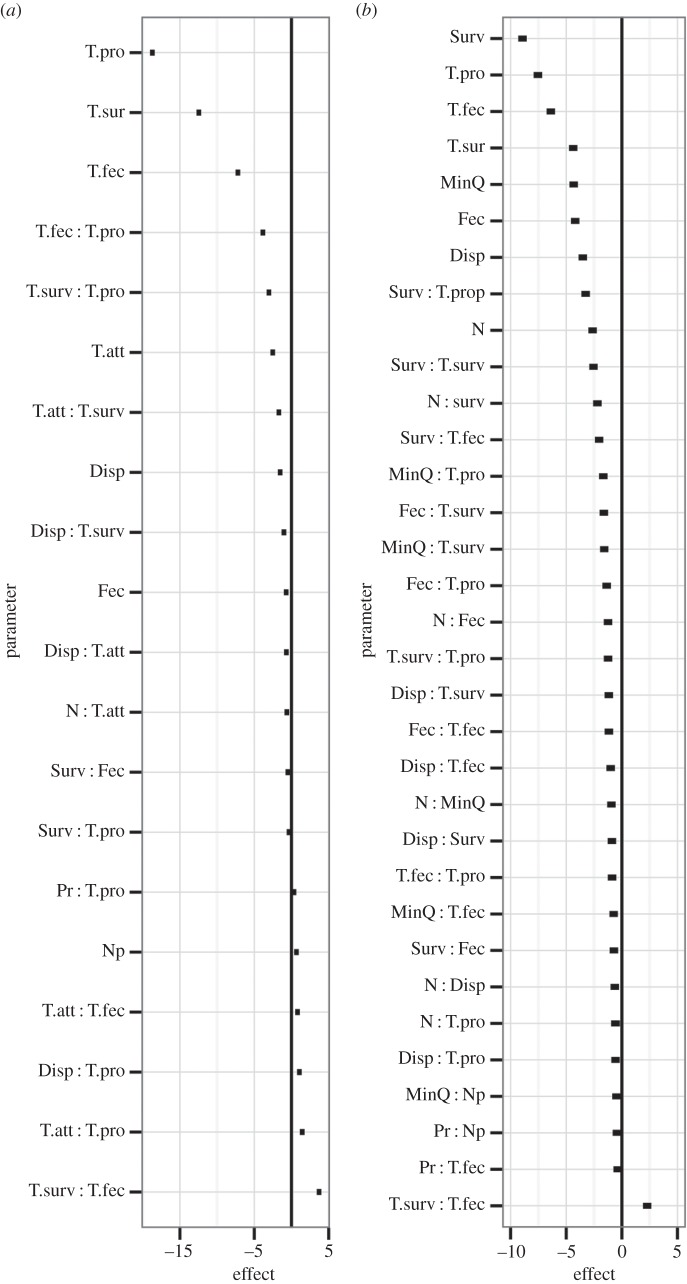
Results of generalized linear model (GLM) describing the relative influence of variables on differences in metapopulation (*a*) growth rate (*λ*_M IMPACT_) and (*b*) mean lifetime (MMLT_IMPACT_) between metapopulations with and without ecological traps. Bars show 95% CIs associated with slopes and interactions between slopes. The *x*-axis describes the effect of a one standard deviation change in each model parameter on the response. Parameters (statistically significant at *p* < 0.05) are organized according to the direction and strength of their influence on the response. Overall fits (*R*^2^) of GLM: (*a*) 0.91 and (*b*) 0.77. Model parameters are defined in table 1.

All criteria in our framework influenced the negative consequences of traps. The proportion of traps (T.pro) in the landscape was a strong influence on both *λ*_M IMPACT_ and MMLT_IMPACT_ (figures 2 and 3), and is likely to be the most important determinant of whether animals encounter traps. We found some evidence to suggest that highly vagile species (i.e. higher Disp) may also be more susceptible to the effects of traps, but perceptual range was less important.

**Figure 4.**
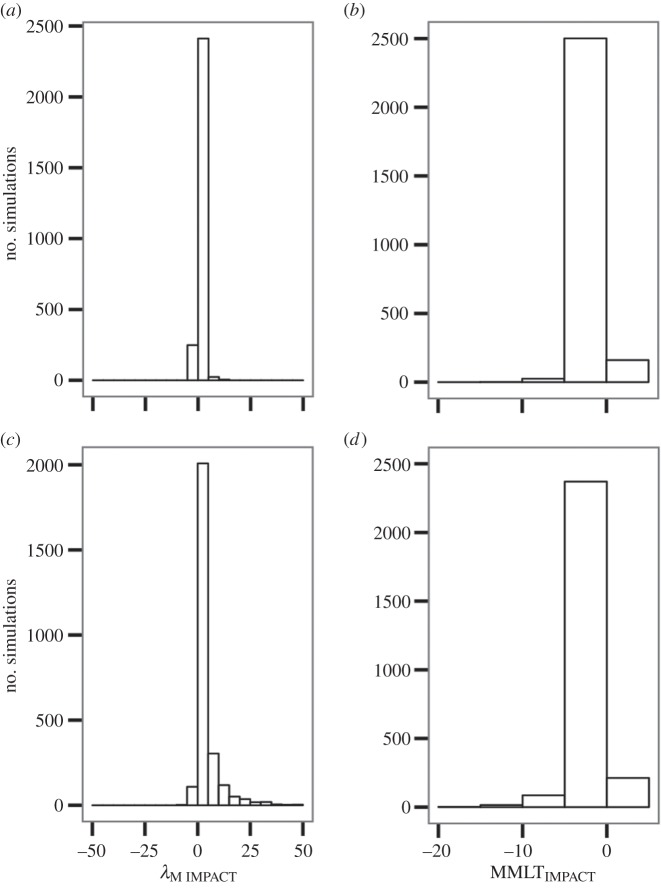
The influence of natal habitat preference induction (NHPI) on metapopulations with and without ecological traps. The four panels illustrate the effects of NHPI on *λ*_M IMPACT_ and MMLT_IMPACT_ when ecological traps (*a*,*b*) are and (*c*,*d*) are not present in the landscape. There were 12 simulations where *λ*_M IMPACT_ was greater than 50 when traps were present (*c*); these cases are not shown, to allow a clearer presentation of overall results.

The probability of animals selecting traps is likely to be influenced by their attractiveness relative to other habitats. More dramatic impacts were observed on *λ*_MIMPACT_ when the attractiveness of traps (T.att) was higher relative to non-traps. In comparison, trap attractiveness was not important to MMLT_IMPACT_ (figure 2*b*). NHPI was not one of the most important factors influencing the consequences of traps, especially for MMLT_IMPACT_ (Np, figures 2*b* and 3*b*). However, our GLM suggested that Np weakened the effects of traps on *λ*_M IMPACT_ (figure 3*a*). When landscapes did not contain traps, Np had only relatively weak effects on MMLT_IMPACT_ and *λ*_MIMPACT_ (figure 4*a,b*). In comparison, Np generally resulted in stronger positive effects on *λ*_MIMPACT_ values when traps were present (figure 4*c*). We also observed instances, though, where Np magnified the effects of traps on *λ*_MIMPACT_ values.

Reductions in fecundity (T.fec) and survival (T.surv) in traps negatively affected both metapopulation responses, and for MMLT_IMPACT_ the magnitude was comparable. However, reduced survival in traps had a stronger negative effect on *λ*_M IMPACT_ than reduced fecundity. The positive interaction between the two parameters (T.fec : T.surv; figure 3) reflects the fact that as one parameter increases, the relationship (i.e. slope) between the other parameter and the metapopulation impact increases (or becomes less negative). For example, with a low survival penalty, the relative impact of a high fecundity penalty is quite significant, whereas at high survival penalties, the impact resulting from increasing the fecundity penalty is reduced.

In general, a species's intrinsic survival and fecundity had negative influences on MMLT_IMPACT_ and *λ*_MIMPACT_ (Surv and Fec; figures 2 and 3). Model species with higher survival suffered greater (i.e. more negative) metapopulation impacts resulting from ecological traps. Increases in survival and fecundity increased MMLT and *λ*_M_ in the absence of traps, but this did not translate into a decreased impact in the presence of traps, as expected. Thus, for a given trap penalty, species with higher fitness will have a greater absolute decrease in reproductive success/survival in trap patches than those species with lower fitness.

## 4. Discussion

All criteria in our framework significantly influenced the negative consequences of traps, illustrating that assessing traps requires an approach integrating landscape and movement ecology, habitat selection behaviour, and the life history of animals. We evaluate the evidence below for our six hypotheses to assess their relative influence on the consequences of traps for metapopulation dynamics.

**Hypothesis 1.**

The effects of traps will be more pronounced when they represent a larger proportion of available habitats.

The proportion of traps (T.pro) was a key determinant of how traps reduce both metapopulation growth rate and mean lifetime. Previous models have suggested there may be a threshold proportion of traps in the landscape above which extinction probability increases, potentially dependent on habitat quality and preferences [9,12]. For example, migratory songbirds are likely to suffer population extirpations when traps represent more than 30% of available habitats [9]. We observed that *λ*_M IMPACT_ and MMLT_IMPACT_ were increasingly negative when T.prop was high, but found no evidence that similar threshold effects occurred when all other habitat attributes (quality, size, placement, etc.) varied continuously across the landscape.

**Hypothesis 2.**

The chances of an animal encountering a trap will depend on their dispersal ability or perceptual range.

Highly dispersive animals may be able to rescue or recolonize patches and thus be less predisposed to extinction risk in fragmented landscapes [51]. Our results illustrate an opposing perspective, with highly vagile species more susceptible to the impact of ecological traps. Our GLM results provide some support for three possible reasons for this effect: (i) an increased rate of trap encounter (Disp : T.att, Disp : T.pro, figure 3*a*; Disp : T.pro, figure 3*b*), (ii) a greater neighbourhood (i.e. number of habitat patches) that trap patches influence (N : Disp, figure 3*b*) or (iii) a decrease in the local retention of offspring in quality habitat with increased dispersal (see Disp formulation in table 1). The ultimate cause may be a combination of these, in addition to other potential costs of dispersal [52].

While increased perceptual range (Pr) may lower dispersal costs and increase connectivity in fragmented landscapes [53], the opposite has also been shown. For example, simulations studies have shown that animals with no perceptual knowledge of their environment had increased overall metapopulation connectivity in comparison with those with increased cognitive information, at least while energy resources where adequate [54]. We found similar effects, with increased perceptual range having no influence on the impact of traps. The influence of Pr on metapopulation dynamics and the impact of traps may be masked by the randomized spatial structure of our landscapes, which had a strong influence on metapopulation connectivity. On average, the spatial structure of habitat patches and the species' dispersal potential (Disp) accounted for 84% of the metapopulation connectivity matrix, the remaining proportion determined by the perceptual range, cue distances and attractiveness (gravity) of patches in the model.

**Hypothesis 3.**

Severe (i.e. preferred) traps will reduce metapopulation growth rate and persistence more than equally preferred traps.

Severe traps are likely to lead more frequently and rapidly to the decline and extirpation of animal populations as individuals are attracted away from higher fitness habitats [10,11]. Our results indicate severe traps may similarly have more dramatic consequences than equal-preference traps for metapopulations. In comparison, trap attractiveness was not important to MMLT_IMPACT_. This lack of impact was found across all meta-models, suggesting its effects may be somewhat obscured by the strong (and uncontrolled) effect of network topology [20].

**Hypothesis 4.**

Animals that exhibit NHPI will be more likely to select traps.

Our results illustrate that NHPI has a strong influence on habitat selection when traps are present in the landscape, and that generally this resulted in the effects of traps being diluted. Kokko & Sutherland [11] proposed that preferences for natal-like habitat may provide some protection from the effects of traps as the increased productivity of high-quality habitats means that more individuals will be selecting these over poorer-quality options. However, our results also illustrate that NHPI could magnify the effects of traps in some cases (figure 4*c*). Recent evidence suggests that NHPI does not always lead to the selection of highest-quality habitats (e.g. [29]), and could lead to traps, if for example habitats are exposed to pollutants that go undetected and animals continue to select polluted sites (e.g. bats foraging on non-biting midges associated with sewage effluent [55]). If so, NHPI could, in rare instances, facilitate the initial development of traps and their subsequent persistence through a negative feedback loop where individuals continue to select impacted environments.

**Hypothesis 5.**

Reductions in breeding fitness and mortality will have differential effects.

Studies of traps at the local scale (e.g. those reviewed in [4]) have illustrated how traps may reduce fitness, for example, by characterizing rates of survival or breeding success. Extending this finding to the landscape level, we have modelled how local reductions in fitness affect metapopulation growth and persistence. Our results demonstrate that reduced fecundity (T.fec) or survival (T.sur) in traps resulted in comparable reductions in metapopulation persistence. However, metapopulation growth rate is likely to be more limited when traps reduce survival compared with fecundity. The interaction we observed (T.fec : T.surv) illustrates that when traps reduce one of these elements of fitness, the overall effects are not exacerbated by subsequent reductions in the other.

**Hypothesis 6.**

Animals with life-history traits from the ‘fast’ end of the fast–slow continuum will be more susceptible to traps.

Our results illustrate that animals with high intrinsic fitness are likely to be more susceptible to the effects of traps, based on having more scope for negative effects to occur. However, species with ‘slow’ life-history traits will be more likely to suffer local extirpations—high intrinsic fitness may mean that traps can result in larger reductions in fitness, but may also confer increased resilience to traps.

In modelling the consequences of ecological traps on metapopulations, we focused on survival and fecundity to estimate the life-history traits of animals likely to influence their susceptibility to traps. However, other life-history traits (e.g. electronic supplementary material, appendix S1) will probably influence how animals respond once trapped; for example, those traits that facilitate rapid evolution may offer the potential for animals to ‘escape’ via natural selection for adaptive preferences or existing phenotypic plasticity [11]. Other traits, such as those that influence the evolution of dispersal ability, will potentially also be important. A logical extension to our approach here would be to examine the influence of some of these evolutionary traits on metapopulation growth rate and persistence when traps are present in landscapes.

### (a) The triple jeopardy of ecological traps: prevalence, attractiveness and fitness consequences

Complex interactions between the spatial arrangement of traps, their attractiveness and fitness costs, and the life-history traits of animals will ultimately determine how metapopulations respond to traps. However, it is clear that animals are likely to be most at risk when traps occur under the ‘triple jeopardy’ scenario, whereby they (i) are highly attractive, (ii) result in large reductions in fitness and (iii) represent a large proportion of the available habitat. These observations are intuitive, and similar suggestions have been made about the effects of traps at local scales [11,13], but our study provides the first evidence that they still hold at metapopulation scales.

### (b) Ecological traps may be beneficial in rare instances

We present the first evidence that in rare instances, traps may have positive benefits for metapopulations. While traps generally had negative effects, in a small number of situations they increased metapopulation growth rate (positive *λ*_MIMPACT_). This occurred primarily when traps, characterized by increased attractiveness and only minor fitness costs, served as central stepping stones in a habitat network, effectively increasing landscape-scale connectivity as animals move through the trap patch. This benefit was confirmed through a targeted modelling ensemble. A 19× increase in the likelihood of a positive *λ*_M IMPACT_ value was achieved by modelling vagile taxa in a high-quality landscape where the proportion of traps was low (less than 30%), the trap attractiveness was high (more than 5×) and the fitness consequences were low (penalties < 0.10). We suspect this effect is strongly dependent on the topology of patches and the placement of traps.

### (c) The impact of naturally occurring trap-like patches

Our results suggest that trap-like conditions are probably a common phenomenon of metapopulations in landscapes where patches vary in quality and size. More than 87% of simulations contained naturally occurring trap-like patches (electronic supplementary material, appendix S2), and these represented on average approximately 38% of patches across all simulations. Results from running a targeted global SA with the proportion of natural trap-like patches as a parameter illustrate that while these patches may be common, their effects in the presence of ecological traps are weak (relative influence 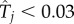). With the prevalence of naturally occurring trap-like patches within spatially realistic metapopulations, we suggest clarity is needed in future studies to distinguish between these patches and ecological traps where habitat selection cues and/or habitat quality have been altered.

## 5. Conclusion

Evaluating the risks ecological traps pose to animal populations requires a greater understanding of their impacts within the landscape. By developing a generalized spatial framework, we have shown that the severity of traps depends not just on their fitness consequences, but also the life-history traits of animals. In particular, traits that increase the likelihood of encountering and selecting traps, as well as a species's vulnerability to the associated fitness costs, are likely to be important. Our findings further demonstrate that the effects of traps become significantly more complicated when the focus is on landscape rather than local scales, requiring a broader consideration of how animals move across spatially heterogeneous landscapes. A crucial next step to further improve our understanding is to use our findings to develop and test predictions about the effects of traps on metapopulations in the field.

Ecological traps are likely to become increasingly common as humans continue to dramatically alter the landscape, and therefore have important implications for the management of animal populations worldwide. Incorporating traps into management and conservation practices will require close tracking of changes in both ‘real’ and perceived habitat quality over time, and a greater consideration of animal behaviour [4]. At the local scale, either increasing the quality or decreasing the attractiveness of traps will reduce their effects on animals, but, as our study highlights, managing their effects at the landscape scale is likely to be significantly more complex. Habitats need to be managed within the context of landscape mosaics and the entire landscape [56], rather than at the scale of habitat patches. There is an urgent need, therefore, to assess how traps fit within the gradients of habitat quality that occur in the face of anthropogenic disturbances to the landscape, and to use this broader perspective as the basis for minimizing their effects on animal populations.

## Supplementary Material

Appendices S1-S7
