# Supplementary material for: Evaluating the metapopulation consequences of ecological traps
Source: Proc Biol Sci. 2015 Apr 7;282(1804):20142930. doi: 10.1098/rspb.2014.2930 (PMC4375870; doi:10.1098/rspb.2014.2930)
Supplement: Appendices S1-S7 [file rspb20142930supp1.docx]

**Appendices**

**S1. Characteristics of animals that are likely to increase the probability of encountering and selecting an ecological trap, and species-specific vulnerability to the fitness costs of traps.** Proposed by: ^1^Battin (2004) and ^2^Kokko and Sutherland (2001).

| **Likelihood of encountering (Criteria 1) and selecting (Criteria 2) a trap** | |  | **Species-specific vulnerability to fitness costs of traps (Criteria 4)** | |
| --- | --- | --- | --- | --- |
| ***Characteristics of animals*** | ***Traits likely to increase susceptibility*** |  | ***Characteristics of animals*** | ***Traits likely to increase severity*** |
| Dispersal | - Range-restricted^1^ - Dispersal associated with habitat transitions that are an obligate part of the life cycle - High vagility may increase encounter rates (but also provide greater capability of ‘escaping' traps) - Low vagility may reduce capacity for redispersal after encountering traps (but encounter rate may be decreased) - Large perceptual range may result in detecting traps from greater distances |  | Adaptive potential | - Slow rate of evolution^1^ - Low capacity for learning^1^ - No behavioural adaptations to change^1^   Adaptive potential may be a possible mechanism rescuing animals from traps^2^ |
| Habitat selection behaviour | - Reliance on simple cues- easier to decouple habitat choice from quality - Reliance on indirect cues^1^ - Specialists on trap habitat - Imperfect knowledge of environment^1^ - Assess habitats on absolute (i.e. is a habitat good/poor) rather than relative (i.e. how does habitat A compare to habitat B) criteria - Low within-population variation in habitat selection traits^1^ - Site-attached after dispersal - Habitat selection leads to close association with stressor (e.g. benthic organism and sediment pollution) |  | Reproduction | - Small population size^1^ - Low fecundity - Long reproductive cycle - Semelparous breeding- no opportunity for experience-based learning |
|  |  |  |  |  |

**S2. Detailed description of modelling approach.**

Each metapopulation simulation (with N subpopulations) was initialized by randomly assigning habitat patch attributes (location, size, and quality) for all patches in the 100 x 100 unit landscape. The maximum size allowed for all habitat patches was restricted so that at most 40% of the landscape could be filled, guaranteeing a patchy habitat structure. The quality of each patch was randomly chosen with a minimum quality set by the parameter MinQ. Patch locations were reassigned, if needed, to ensure habitat patches did not overlap. Independent to the landscape characteristics, species-specific attributes were assigned for dispersal or movement capabilities (Disp), perceptual range (Pr), patch preference or attractiveness (a function of patch size, quality, and Pr), as well as survival (Surv) and fecundity (Fec). From this unaltered landscape, a proportion of habitat patches (T.prop) was then selected as potential ecological traps. For these trap patches, survival and fecundity were decreased (by T.surv, T.fec penalties) and the perceptual range increased by the trap attractiveness multiplier (T.att).

Demonstrating an ecological trap requires three conditions to be met: (1.) individuals exhibit a preference for one habitat over another (a “severe” trap), or equal preferences for both habitats (an “equal-preference” trap), (2.) fitness differs between habitats, and (3.) the fitness outcomes for individuals settling in preferred or equivalent habitats (depending on if the trap is severe or equal preference) is lower than in other available habitats (Robertson & Hutto 2006). Consequently, each potential trap within a simulation was compared to its neighbours (defined as any patch within a dispersal probability of 0.0001 of the focal patch) and was considered a realised trap if it had a lower fecundity and/or lower survival and was equally or more attractive than at least one neighbour. If a potential trap was not a realised trap, its patch attributes were returned to the unaltered landscape state. An example landscape configuration and summary of our code are provided in Appendix S4 and S5.

Similar to other spatially-realistic metapopulation models, our landscapes also included naturally occurring patches with trap-like attributes that meet the criteria of an ecological trap without any manipulation of their quality or attractiveness (illustration in Appendix S5). We assumed that animals did not have a perfect knowledge of the environment (i.e. not leading to an ideal free distribution - Fretwell & Lucas 1970; Fretwell 1972), and consequently, habitat selection was determined by inter-patch distance, and patch size and quality, resulting in some individuals colonising suboptimal habitats. Our interest was in the ecological traps whose characteristics (e.g. habitat quality and attractiveness) had been manipulated to reflect some level of environmental or anthropogenic disturbance, so these naturally occurring trap-like habitat patches were not considered to be realised ecological traps in our analysis.

Dispersal among patches was initially based on a species-specific negative exponential decay function (Urban & Keitt 2001), and then modified using the production-constrained gravity model (Muirhead & MacIsaac 2011) to redistribute individuals based on the attractiveness of the destination patch. Simply, this gravity-based dispersal probability is calculated by multiplying the distance-based dispersal probability by the reproductive output of the source patch, multiplied by the attractiveness of the destination patch (the gravitational pull). This matrix is then row-normalised, each element representing the probability of movement from the source patch (matrix row) to the destination patch (column), accounting for all landscape, species-level, and trap attributes (see Appendix S5 for example matrices). The natal preference penalty (Np) was then used to modify this dispersal probability matrix, redistributing individuals to patches with similar qualities. Due to the unknown prevalence and strength of NHPI, we analysed each metapopulation model with and without the natal preference penalty. The metapopulation consequences of ecological traps were then quantified by comparing the unaltered or non-trap metapopulation model with the model containing ecological traps, as described above, using the metapopulation mean lifetime and the metapopulation growth rate. Therefore, four ‘versions’ of every metapopulation model were evaluated: with and without ecological traps; and with and without the natal preference penalty (Np). There was a high congruence between metapopulation analyses with and without Np (for all analytical methods, Pearsons R > 0.99 comparing the importance of all parameters with and without Np), so we present results in the manuscript from comparisons using metapopulation models including Np.

Four meta-models were chosen for the global sensitivity analysis (SA) as each is expected to perform differently depending on the unknown structure of the response surface. The GLM was included as it is a common choice in ecology and conservation, is very fast, and is straightforward to implement and interpret (Coutts & Yokomizo 2014). For the GLM SA, we calculated the main effects and two-way interactions on the standardized data using the identity link function, and visualised the sensitivity of response variables to the model parameters by plotting the effect of one standard deviation change in each parameter on the response (Coutts & Yokomizo (2014). The QRS includes stepwise variable selection and will typically outperform other more complex models when a quadratic function best approximates the response surface (Storlie & Helton 2008). Recursive partitioning (TREE) makes few assumptions regarding the data and should perform well particularly when discontinuities exist in the response surface. MARS, a more recent approach, combines spline regression, recursive partitioning, and variable selection to quantify the response surface (Storlie & Helton 2008). MARS is expected to outperform many other meta-models when the response surface is smooth.

**S3. Selection of T.fec, T.surv and T. att ranges for metapopulation modelling.**

To develop realistic ranges for the likely attractiveness (i.e. strength of preference) for traps, we reviewed studies of ecological traps cited in Robertson et al. (2013). Focussing only on those studies where the effects of an ecological rather than evolutionary trap were studied and information on effect sizes was available (n=14), we calculated the attractiveness as: abundance in traps/abundance in non-trap habitats (or experimental preference for traps/non traps if this was studied). In these studies, the mean attractiveness was 6 (standard error = 3.2), ranging from 1 (i.e. an equal preference trap) to ~40. Our range [0,10] was selected to reflect a realistic range of likely trap attractiveness values.

We used ranges of [0,1] for T. surv and T. fec to reflect that traps could result in complete mortality or reproductive failure. This is justified based on previous studies where these drastic effects have been documented, for example, dragonflies ovipositing on crude oil or artificial substrates (Kriska et al. 1998, Horvath et al. 1998, Horvath et al. 2007).

**S4. Pseudocode for metapopulation modelling to examine landscape scale consequences of ecological traps.**

For each of the 3,000 combination of parameters {

calculate maximum size of patches, **maxS**

randomly assign patch coordinates (**x**,**y**), **size**, and quality, **q**, for patches

(check for patch overlap and reassign x, y, size, if needed)

For all N habitat patches {

calc detection distance, **CueDist_i_** as **Pr** * **qi** * radius_i_

calc survival, **s_i_** as **Surv** * **q_i_**

calc fecundity, **f_i_** as **Fec** * **q_i_**

calc all pairwise centroid-to-edge distances as symmetric matrix, **D**

convert **D** to a probability matrix, **P**, using **Disp**

randomly select **T.prop** * **N** patches as traps

For all Trap patches reassign patch attributes {

quality **q_i_** as **q_i_** * (1-**T.surv***0.5) * (1-**T.surv***0.5)

survival **s_i_** as **s_i_** * (1-**T.surv**)

fecundity **f_i_** as **f_i_** * (1-**T.fec**)

attractiveness (detection limit), **CueDist_i_** as **CueDist_i_** * **Trp.att**

calc gravity-based dispersal probability, **gP_ij_** = **C_i_** * **RO_i_** * **W_j_** * **P_ij_**, where

**C_i_** = row normalisation ‘balancing factor’

**RO_i_** = reproductive output of source as **f_i_** * **size_i_**

**W_j_** = attractiveness of destination as area of **CueDist_i_**

**P_ij_** = distance-based dispersal capacity

modify gP_ij_ with natal preference penalty as ***p_ij_*** * (1-(|***q_i_*** - ***q_j_***| * **Np**))

identify realized traps: if initial trap has (lower fecundity OR

lower survival) AND greater attractiveness than neighbours

(those with a pij > 0.0001). If not a realised trap, then

revert patch attributes back to non-trap state

calc metapopulation mean lifetime, **MMLT** (code from Kininmonth et al. 2010)

MMLT = ƒ(**gP**, **RO**, η = 0.10, ε = 45, μ = 2), where

η is a scaling parameter related to environmental variation

ε is the area coefficient for the local extinction rate

μ is the minimum number of immigrants needed

calc metapopulation growth rate, **LambdaM** (from Figueira & Crowder 2006)

LambdaM = ƒ(**gP**, **RO**, **S**) where

**S** is a vector of survival in each patch, **s_i_**

calc MMLT in the absence of traps and natal preference, **MMLT.null**

calc LambdaM in the absence of traps and natal preference, **LambdaM.null**

calc Trap impact, **MMLT.delta** = ((log(MMLT_Trap_+1) – log(MMLT_non-Trap_))/log(MMLT_non-Trap_))*100

calc Trap impact, **LambdaM.delta** = ((LambdaM_Trap_ – LambdaM_non-Trap_)/LambdaM_non-Trap_)*100

**S5. Metapopulation model illustration and data.**

Below we present an illustration of one particular landscape, similar to those explored in our modelling framework. Shown are plots of the spatial structure of patches and perceptual range (right), the non-trap (top) and trap-based (bottom) connectivity matrices, and the model parameters, patch-level attributes, and model output. Comparing patches 1, 3 and 4 provides an illustration of where a naturally occurring habitat patch can have the trap-like characteristics described in the manuscript, as patches 1 and 3 are more attractive (O.CueDist) than patch 4, but are of lower quality (O.qual), and subsequently have lower associated fitness (O.surv and O.fec). None of these naturally occurring patches were treated as ‘realised ecological traps’ in our modelling.

**
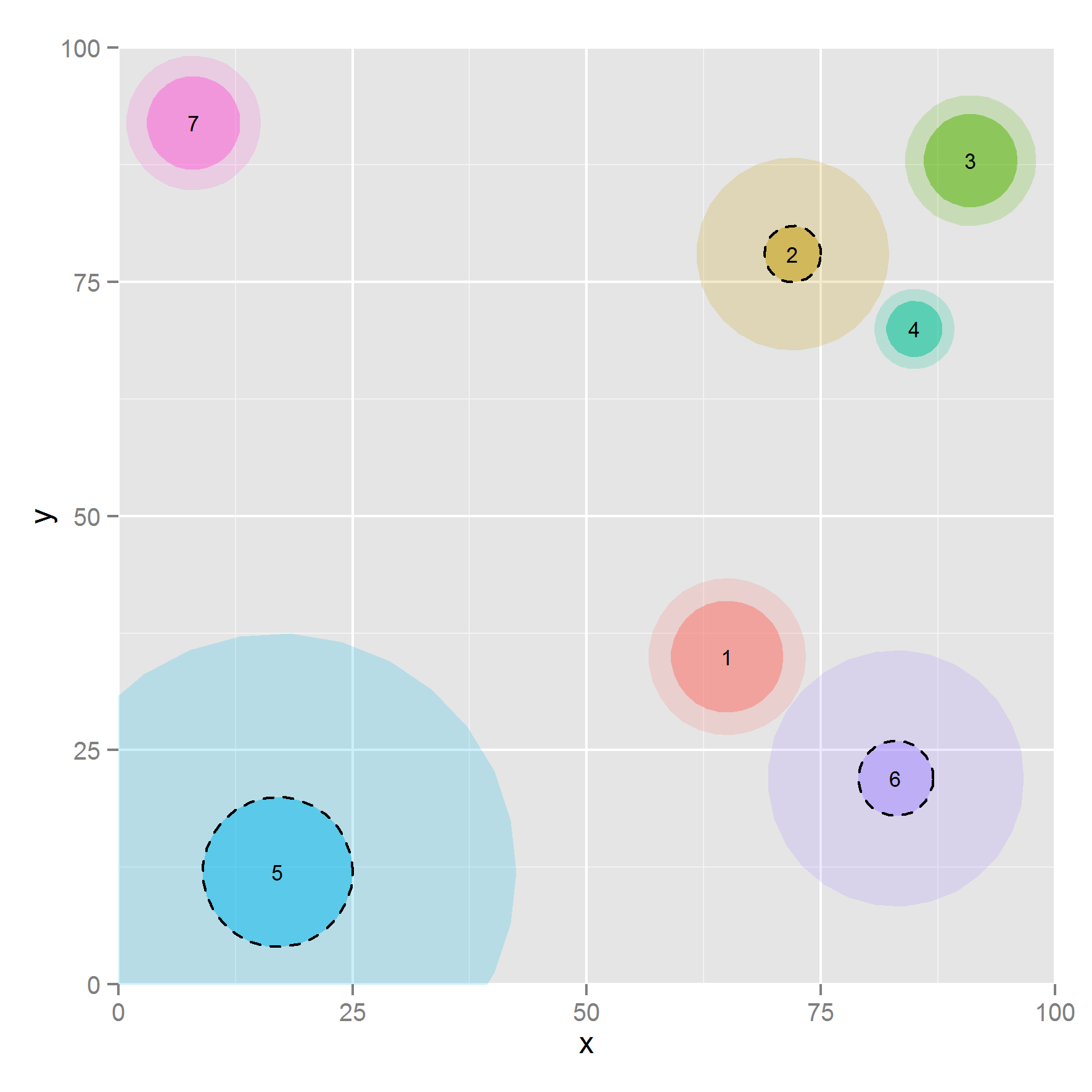
gP (Non-trap model gravity-based dispersal probability):**

|  |  | **Destination** | | | | | | |
| --- | --- | --- | --- | --- | --- | --- | --- | --- |
|  |  | **1** | **2** | **3** | **4** | **5** | **6** | **7** |
| **Source** | **1** | 0.885 | 0.004 | 0.002 | 0.006 | 0.012 | 0.091 | 0.000 |
|  | **2** | 0.032 | 0.506 | 0.280 | 0.179 | 0.000 | 0.002 | 0.002 |
|  | **3** | 0.004 | 0.061 | 0.847 | 0.088 | 0.000 | 0.001 | 0.000 |
|  | **4** | 0.042 | 0.146 | 0.331 | 0.476 | 0.000 | 0.006 | 0.000 |
|  | **5** | 0.006 | 0.000 | 0.000 | 0.000 | 0.994 | 0.000 | 0.000 |
|  | **6** | 0.340 | 0.001 | 0.001 | 0.003 | 0.003 | 0.652 | 0.000 |
|  | **7** | 0.000 | 0.000 | 0.000 | 0.000 | 0.001 | 0.000 | 0.998 |

**gP (Trap model gravity-based dispersal probability):**

|  |  | **Destination** | | | | | | |
| --- | --- | --- | --- | --- | --- | --- | --- | --- |
|  |  | **1** | **2** | **3** | **4** | **5** | **6** | **7** |
| **Source** | **1** | 0.679 | 0.010 | 0.001 | 0.004 | 0.036 | 0.269 | 0.000 |
|  | **2** | 0.006 | 0.904 | 0.053 | 0.031 | 0.001 | 0.004 | 0.000  **Example landscape** where dark centres represent the suitable habitat patches and the transparent region is the distance at which patches can be detected (perceptual range). Traps are shown with dashed borders.  **Input parameters**: N=7, MinQ=0.5, Pr=0.5, Surv=0.9, Fec=2, Disp=0.25, Np=0.5, T.prop=3/7, T.att=6, T.fec=0.9, T.surv=0.9.  **MMLT_IMPACT_** = -14%  **λ_M IMPACT_** = -21% |
|  | **3** | 0.003 | 0.199 | 0.724 | 0.071 | 0.000 | 0.002 | 0.000 |
|  | **4** | 0.029 | 0.378 | 0.230 | 0.348 | 0.001 | 0.015 | 0.000 |
|  | **5** | 0.001 | 0.000 | 0.000 | 0.000 | 0.999 | 0.000 | 0.000 |
|  | **6** | 0.052 | 0.001 | 0.000 | 0.000 | 0.005 | 0.941 | 0.000 |
|  | **7** | 0.000 | 0.001 | 0.000 | 0.000 | 0.002 | 0.000 | 0.996 |

**Patch attributes (O. = pre-trap values; T. = Trap values):**

| ID | x | y | Trap | size | O.qual | O.surv | O.fec | O.CueDist | T.qual | T.CueDist | T.surv | T.fec |
| --- | --- | --- | --- | --- | --- | --- | --- | --- | --- | --- | --- | --- |
| 1 | 65 | 35 | 0 | 113 | 0.8 | 0.72 | 1.6 | 8.4 | 0.8 | 8.4 | 0.72 | 1.6 |
| 2 | 72 | 78 | 1 | 28 | 0.8 | 0.72 | 1.6 | 4.2 | 0.08 | 10.3 | 0.072 | 0.16 |
| 3 | 91 | 88 | 0 | 79 | 0.8 | 0.72 | 1.6 | 7 | 0.8 | 7 | 0.72 | 1.6 |
| 4 | 85 | 70 | 0 | 28 | 0.9 | 0.81 | 1.8 | 4.3 | 0.9 | 4.3 | 0.81 | 1.8 |
| 5 | 17 | 12 | 1 | 201 | 0.6 | 0.54 | 1.2 | 10.4 | 0.06 | 25.5 | 0.054 | 0.12 |
| 6 | 83 | 22 | 1 | 50 | 0.8 | 0.72 | 1.6 | 5.6 | 0.08 | 13.7 | 0.072 | 0.16 |
| 7 | 8 | 92 | 0 | 79 | 0.9 | 0.81 | 1.8 | 7.2 | 0.9 | 7.2 | 0.81 | 1.8 |

**S6. Representative landscapes with varying degrees of traps.**

Illustrative landscapes showing a range in the proportion and severity of traps and their metapopulation growth rate (λ_M_) and mean lifetime (MMLT). a) No Traps, λ_M_ = 3.658, MMLT = 66.4; b) High proportion of traps (shown in dashed perimeters) with severe penalties, λ_M_ = 1.305 MMLT = 21.3; c) Intermediate proportion of traps with moderate consequences, λ_M_ = 3.5507 MMLT = 58.4; d) High proportion of traps with minor penalties, λ_M_ = 2.592 MMLT = 65.0.


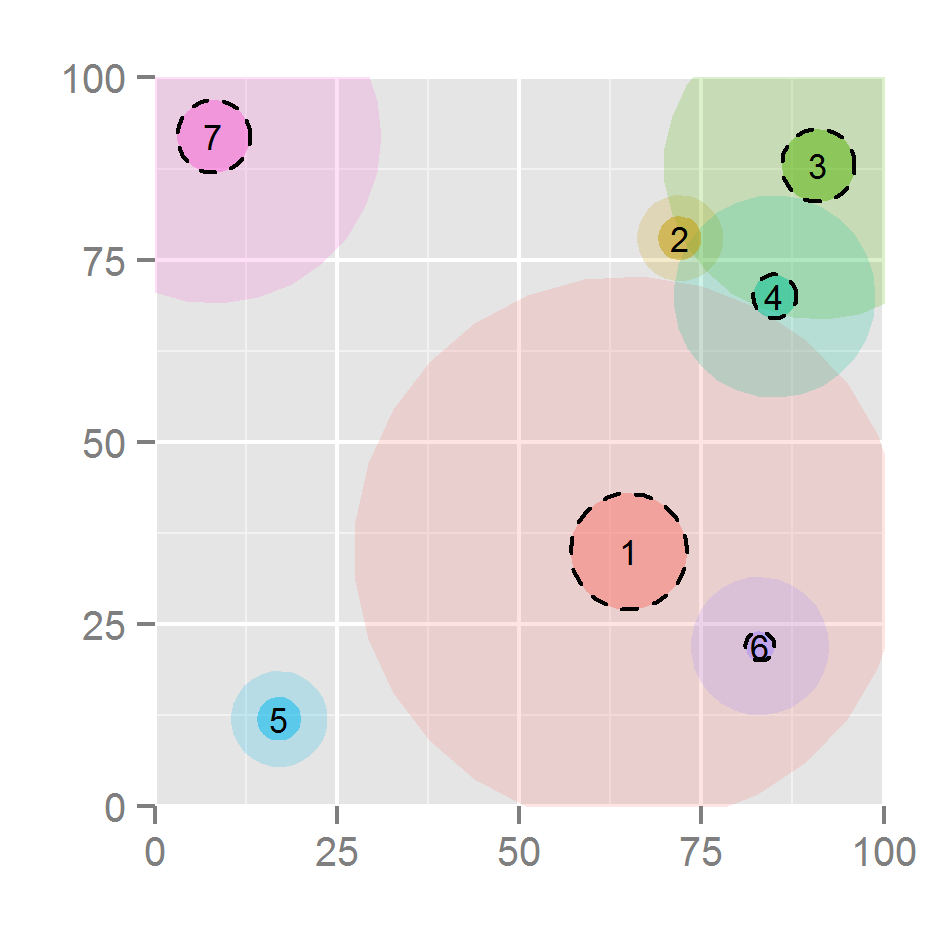

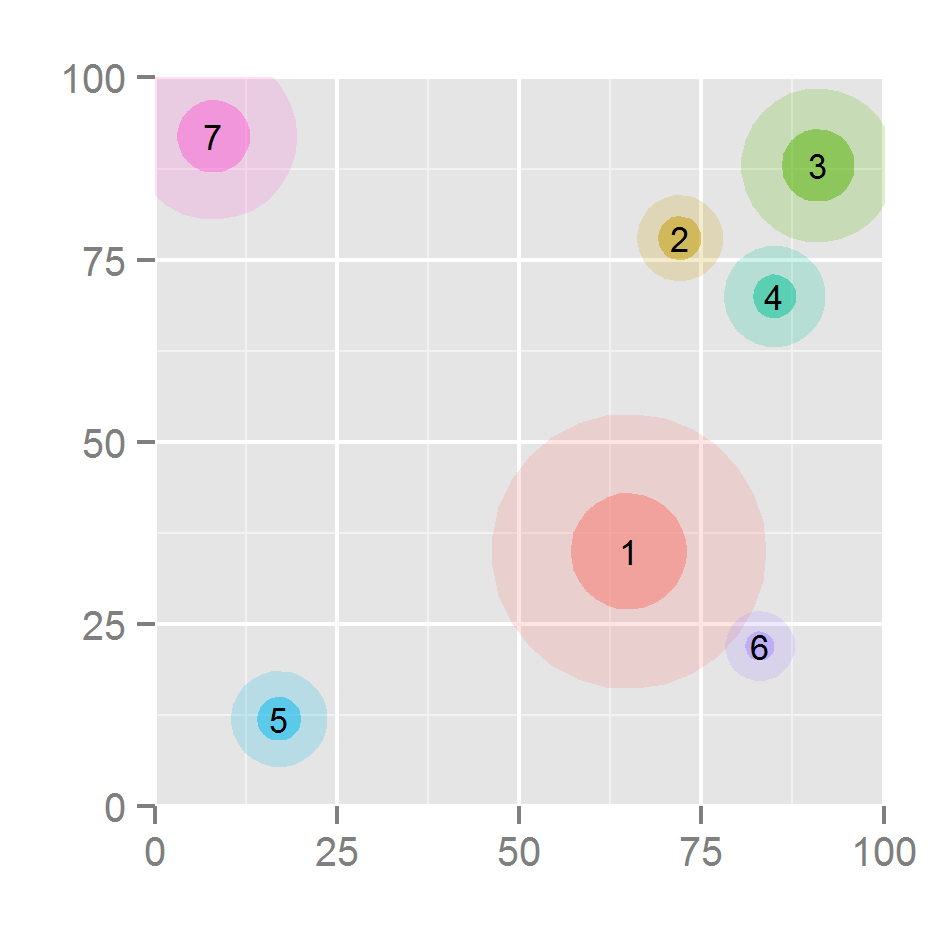


*a)*

*b)*


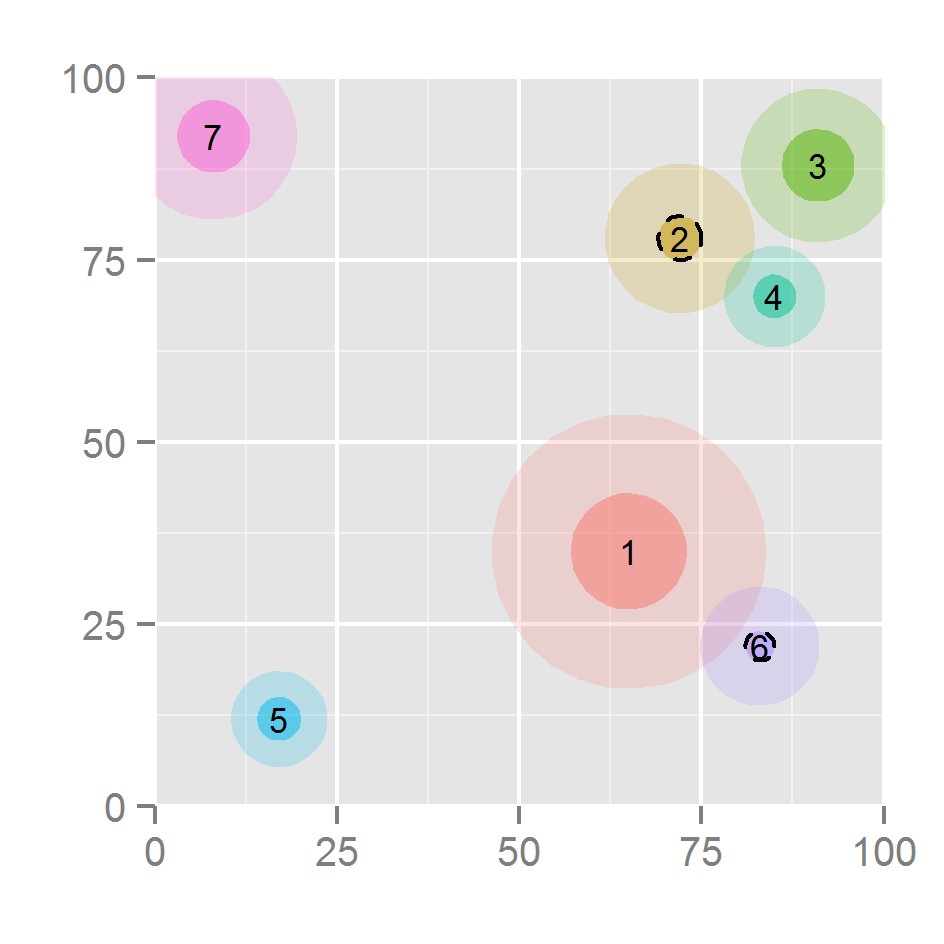


*c)*


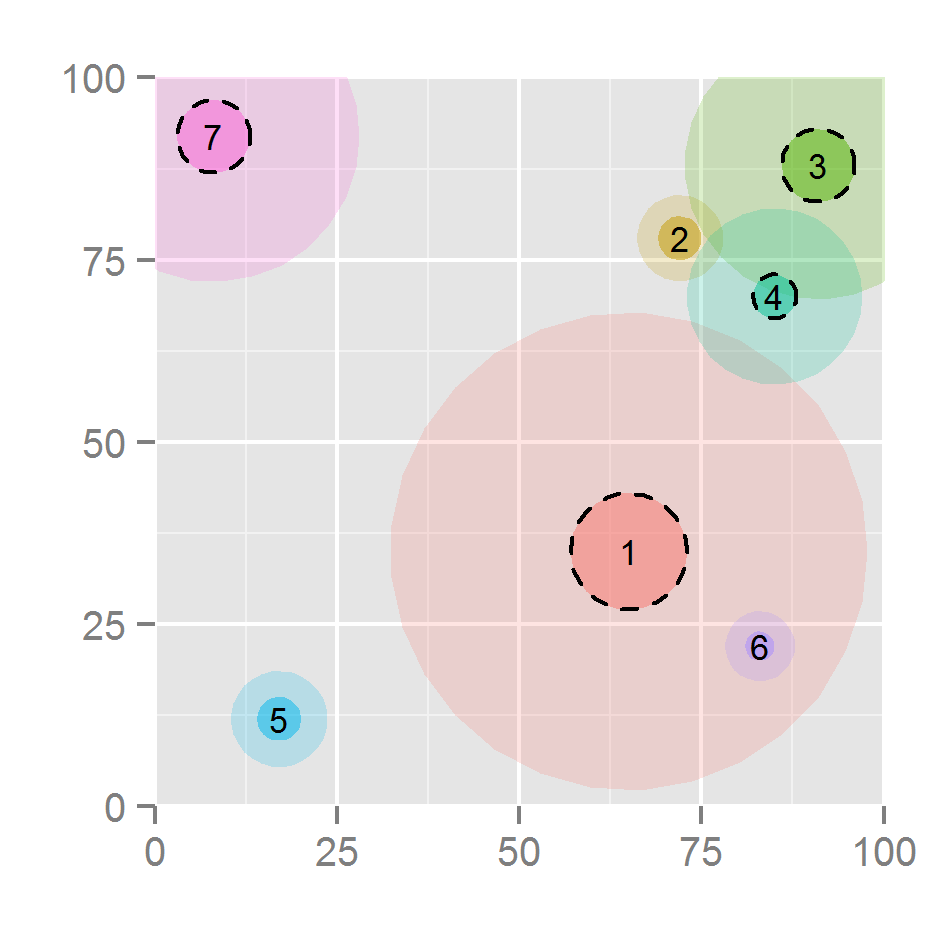


*d)*

**S7. Appendices reference list.**

Battin, J. 2004. When good animals love bad habitats: Ecological traps and the conservation of animal populations. *Conservation Biology* **18**:1482-1491.

Coutts, S. and H. Yokomizo. 2014. Meta-models as a straightforward approach to the sensitivity analysis of complex models. *Population Ecology* **56**:7-19.

Fretwell, S. D. and H. L. Lucas. 1970. On territorial behavior and other factors influencing habitat distribution in birds. I. Theoretical Development. *Acta Biotheoretica* **19**:16-36.

Fretwell, S. D. 1972. Populations in a Seasonal Environment. Princeton University Press, Princeton NJ.

Kokko, H. and W. J. Sutherland. 2001. Ecological traps in changing environments: ecological and evolutionary consequences of a behaviourally mediated Allee effect. *Evolutionary Ecology Research* **3**:537-551.

Horvath, G., Bernath, B. & Molnar, G. (1998). Dragonflies find crude oil visually more attractive than water: Multiple-choice experiments on dragonfly polarotaxis. *Naturwissenschaften* **85**:292-297

Horvath, G., Malik, P., Kriska, G. & Wildermuth, H. (2007). Ecological traps for dragonflies in a cemetery: the attraction of *Sympetrum* species (Odonata: Libellulidae) by horizontally polarizing black gravestones. *Freshwater Biology* **52**:1700-1709

Kriska, G., Horvath, G. & Andrikovics, S. (1998). Why do mayflies lay their eggs en masse on dry asphalt roads? Water-imitating polarized light reflected from asphalt attracts Ephemeroptera. *Journal of Experimental Biology* **201**:2273-2286

Muirhead, J. and H. MacIsaac. 2011. Evaluation of stochastic gravity model selection for use in estimating non-indigenous species dispersal and establishment. *Biological Invasions* **13**:2445-2458.

Robertson, B. A. and R. L. Hutto. 2006. A framework for understanding ecological traps and an evaluation of existing evidence. Ecology **87**:1075-1085.

Robertson, B.A., Rehage, J.S. & Sih, A. (2013). Ecological novelty and the emergence of evolutionary traps. Trends in Ecology and Evolution **28**: 552-560.

Storlie, C. B. and J. C. Helton. 2008. Multiple predictor smoothing methods for sensitivity analysis: Description of techniques. Reliability Engineering & System Safety **93**:28-54.

Urban, D. and T. Keitt. 2001. Landscape connectivity: A graph-theoretic perspective. Ecology **82**:1205-1218.
